# MRI in detecting facial cosmetic injectable fillers

**DOI:** 10.1186/s13005-016-0124-y

**Published:** 2016-09-06

**Authors:** Sigal Tal, Hillel S. Maresky, Theodore Bryan, Ella Ziv, Dov Klein, Assaf Persitz, Lior Heller

**Affiliations:** 1 Department of Radiology, Assaf Harofe Medical Center, affiliated with the Sackler Faculty of Medicine, Tel Aviv University, Ramat Aviv, Tel Aviv, Israel; 2 Plastic Surgery, Assaf Harofe Medical Center, affiliated with the Sackler Faculty of Medicine, Tel Aviv University, Tel Aviv, Israel; 3 Department of Medical Imaging, Sackler Faculty of Medicine, Tel Aviv Universiy, Zeriffin, 70300 Israel

## Abstract

**Background:**

Despite being considered a non-invasive procedure, injections can cause adverse outcomes including infections, overfilling, asymmetry, foreign body granulomas, and reactions that lead to scarring. Complications may be associated with the procedure itself, the physician’s technique, and/or the type of agent injected. In these instances, it is important to be able locate and identify the substance used. This study investigated the viability of using MRI to correctly identify injected substances, their symmetry of distribution, and related complications.

**Methods:**

Fourteen patients with suspected injectable filler complications were identified by our institution’s plastic surgery service. All subjects were scanned with MRI, using highly specific face-oriented sequences at high resolution with small field of view and thin slices across the axial and coronal planes by T1 Dixon non-contrast, T2 Dixon, and T1 Dixon after gadolinium injection. Two independent and blinded radiologists evaluated the images and reported (1) the likely injected substance, (2) symmetry, and (3) complications. These radiological results were compared against clinical data provided by the plastic surgery service.

**Results:**

Ten patients (83 %) presented objective injectable complications: 4 had abscess, 4 granulomata, and 2 had allergic reactions to the injected substance. The Fleiss Kappa for inter-rater agreement on substances was 0.80. Asymmetry was identified in six patients (50 %) with a Kappa between radiology evaluators of 1. MRI characteristics of these common fillers are summarized in table form.

**Conclusions:**

Given the growing awareness among referring physicians of the value of dedicated facial MRI, utilization of this imaging technique may lead to discovery of the injected substance’s true identity, evaluation of symmetry and/or complications.

## Introduction

As the use of cosmetic injectable fillers continues to rise, so does the incidence of complications. Fillers are commonly administered in office settings by non-specialists and are minimally regulated in many countries [1]. In some cases, the identity of the substance may be unknown or misrepresented. Despite being considered a non-invasive procedure, injections can cause adverse outcomes including infections, overfilling, asymmetry, foreign body granulomas, and reactions that lead to scarring [2]. Complications may be associated with the procedure itself, the physician’s technique, and/or the type of agent injected. In these instances, it is important to be able locate and identify the substance used.

## Background

On the molecular level, injectable fillers are designed to last different lengths of time, and can be categorized as short (0–3 months), medium (3–12 months), or long-term (12 months to indefinitely) [3]. Collagen, hyaluronic acid, and silicone are examples of commonly used short, medium, and long-term fillers, respectively. Generally speaking, shorter-term products have greater biocompatibility [4]. Depending on the type of filler used, complication rates range from 3 % to 52 % [5]. In the short term, adverse events can include bleeding, infections, edema, and migration. In the long term, there is a risk of lump formation, granulomas, abscesses, more diffuse edema, and skin discoloration [2]. In these cases, having a reliable way to identify unknown substances prior to surgical treatment would be of great value both medically and legally [6]. Attenuated total reflectance/Fourier transform infrared spectroscopy can accurately identify unknown injectable fillers [6]. However, this method requires surgical specimen collection, which can lead to undesirable scarring. Spectrophotometers are not commonly found in radiology units, and their implementation requires additional cost outlays and training. Thus clearly, a more ubiquitous and less invasive modality would be advantageous.

MRI and CT studies have documented the features of many common injectable fillers and their related sequelae, and can distinguish them from normal tissues [7]. Calcium hydroxylapatite produces low-to-intermediate signal intensity on T1 and T2 [8]. Collagen fillers are low signal on T1 and high signal on T2. Hyaluronic acid has a relaxation time of 600 ms on T2 [7]. Polyacrylamide is high intensity on T2 and low intensity on T1 [8]. Silicone is hyperintense to water on T1 and either hypointense or isointense on T2, depending on the viscosity of the specific product [9]. However, these descriptions are only useful when the identity of the filler is known and there is simply a need for visualization. Girolamo et al. showed that granulomatous reactions to fillers produced subcutaneous contrast enhancement with IV gadolinium, and found that MRI could be used to detect dermal fillers down to a minimum size of 2 mm without reliance on clinical evaluation [5]. Kadouch et al. assessed the extent of agreement between clinical evaluations of injectable filler-related complications and an independent MRI evaluation [10], and found good concordance in uncomplicated cases (85 %), but not in cases with inflammation or migration (32 and 9 %, respectively).

This study investigated the feasibility of using a dedicated imaging protocol for the assessment of all filler-related procedures and related complications, independent of expert clinical input. We show that by the addition of a high resolution face MRI using T1 T2 Dixon with a fat and water suppression technique, radiologists can identify fillers in unexpected locations, and differentiate different kinds of facial fillers.

Based on the descriptions of injectable fillers [7–9], and our own clinical experience using the T1 T2 Dixon fat suppression (FS) technique, we also generate a reference list (Table 1) that summarizes the characteristics of some commonly used substances on T1, T2, T1FS, and T2FS. This table was provided to the two blinded radiologists who took part in the study, and were asked refer to it to determine the type of injectable filler most likely used in the cases submitted to them.

**Table 1:** Dermal filler characteristics on T1 weighted, T2 weighted, T1 Fat Saturation (T1FS), T2 Fat Saturation (T2FS) sequences

Agent	T1	T2	T1fs	T2fs
Polyacrymallde	Hypo-iso	Hyper	Iso-hyper	Hyper
Hyaluronic	Hypo	Hyper	Hypo	Hyper
Collagen	Hypo	Hyper	Iso-hyper	Hyper
Silicone	Hyper	Hypo	Hypo	Hypo

## Methods

### Patient population

A prospective analysis was performed on all patients above the age of 18 who underwent dedicated face MRI after injection of intradermal fillers at our institution between 2013 and 2015. These patients were referred to our imaging department from the plastic surgery department and outpatient clinics as well as from private esthetic clinics after complaining of asymmetry, facial pain, or both.

### MRI technique

All the patients’ faces were examined with a Siemens *Magnetom Skyra* 3 Tesla MRI, using highly specific face-oriented sequences with high resolution and a small field of view (voxel size: 0.3*0.3*4.0 mm – 0.5*0.5*4.0 mm). Thin slices (3–4 mm) across axial and coronal planes were acquired using the following sequences: T1 Dixon fast spin echo (FSE) sequence non-contrast (TR 5610.0 ms, TE 79.0 ms, Flip angle 150 deg), with and without FS, T2 Dixon FSE non-contrast (TR 3800.0 ms, TE 103.0 ms, Flip angle 160 deg), with and without FS, and T1 Dixon FSE after gadolinium injection (TR 576.0 ms, TE 11 ms, Flip angle 131 deg), with and without FS. Axial, coronal and sagital acquisitions were used, with a total scan time of approximately 38 min.

### Imaging evaluation and clinical correlation

Two independent radiologists with 30 years’ combined experience in radiology volunteered to be evaluators, and were blinded to the identity of substances used, the clinical history and information related to the plastic surgeon. They drew conclusions on the identity of the substance, and graded complications and degree of asymmetry (both on a scale of 1–2), based solely on MRI imaging. When disagreements occurred they submitted their conclusions to a third party with 20 years of experience, also blinded, who decided on likely substance identity. Kappa inter-rater tests were conducted for substance identity, degree of complications and degree of asymmetry, and compared to the surgeons’ files.

## Results

Fourteen subjects who underwent MRI using the above protocol took part in the study after signing consent forms. Thirteen females (93 %) and one male (7 %) were examined, with an average age of 46.8 years. The average time between intradermal injection and MRI scanning was 51 days; three patients (22 %) underwent repeat MRI at an average time from filler to MRI of 82 days. Seven patients underwent polyacrylamide gel injection (50 %), three hyaluronic acid (22 %), two silicone (14 %), and two collagen (14 %). The inter-rater agreement between the two evaluators for substance was a high 0.80 using the Fleiss Kappa and after consensus between the two evaluators, the Kappa between imaging and clinical data was 0.96 (near perfect). While polyacrylamide and hyaluronic acid were easily identified (K = 0.86 and 0.84, respectively), silicone and collagen showed greater disparity between the evaluators (K = 0.77 and 0.69, respectively).

MRI detected four cases of (36 %) abscesses, four cases (36 %) granulomata, and three (28 %) cases of allergic reactions to injected substances. Key imaging features of abscesses included ring enhancement after gadolinium injection, while granulomata demonstrated diffuse signal intensity on T1 and T2 without ring enhancement. Allergic reactions were distinguished by the presence of “patchy” signal on all sequences and fat stranding on non fat saturated sequences around the injected substance, without enhancement after gadolinium injection. The Kappa agreement between evaluators for the presence or absence of complications was 1.0 (perfect agreement), and 0.76 for degree of severity. MRI visualized asymmetry in (50 %) of cases and the kappa agreement between evaluators on the presence or absence of asymmetry was 1.0 and 0.72 respectively, with respect to severity.

## Discussion

These results show that facially oriented MRI with contrast is a reliable tool to accurately locate fillers, and identify unintended outcomes such as migration, abscess formation, granuloma, and delayed allergic reaction. The use of a dedicated facial MRI with high spatial resolution and elaborate Dixon sequences not only gives the interpreting radiologist the ability to make a clear-cut decision on the identity of a substance, but also provides the referring plastic surgeon with a better understanding of disease pathology and pathophysiology. In addition to its clinical utility, MRI can provide forensic evidence to medico/legal cases involving injected materials.

**Fig. 1:**
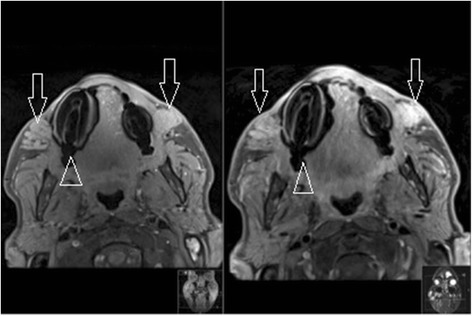
Polyacrylamide gel axial dermal filler appearance in both infraorbital triangles (*open arrows*) on axial MRI (T1FS, T1 FS with gadolinium). Note the susceptibility artifacts from dental implants (*open arrowhead*) while the filler’s signal intensity remains high before and after injection of gadolinium

**Fig. 2:**
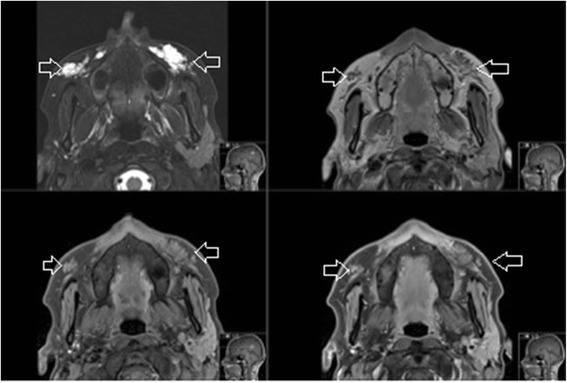
Collagen dermal filler in both infraorbital triangles (*open arrows*) on axial MRI (T2FS, T1, T1FS, T1FS with gadolinium)

Although substances such as silicone and hyaluronic acid have distinct MRI signatures because of their very high or low water content (Fig. 1), intermediate substances tend to produce a more ambiguous image. T1 and T2 Dixon fat suppression with high resolution small FOV MRI make it possible to better visualize and identify these substances (i.e., collagen, Fig. 2). Our original algorithm as summarized in Table 1 which differentiates common fillers in terms of their MRI characteristics, provides a simple guide to assist radiologists in their analysis.

**Fig. 3:**
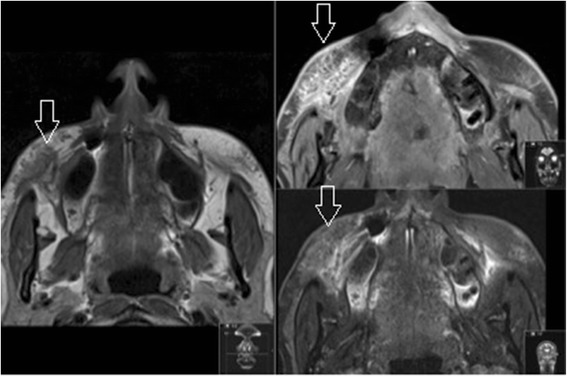
Silicone dermal filler in the right infraorbital triangle (*open arrow*) as seen on axial MRI (T1, T1FS with gadolinium, T2FS). Note how the free silicone oil drops behave like fat on MRI, including a intermediate to low signal on fat suppressed sequences

Dermal fillers are regulated by the Food and Drug Administration in the US, but are only considered medical devices in the UK and elsewhere [11]. Beauticians and other unlicensed individuals can legally administer dermal fillers [12], and the records of these procedures may be unreliable or even nonexistent. Online vendors sell products that may be counterfeit or expired, and therefore of dubious quality [13]. In this risky marketplace, MRI can serve a role both medically and legally (an example of a "dubious" injection discovered to be silicone in Fig. 3).

**Fig. 4:**
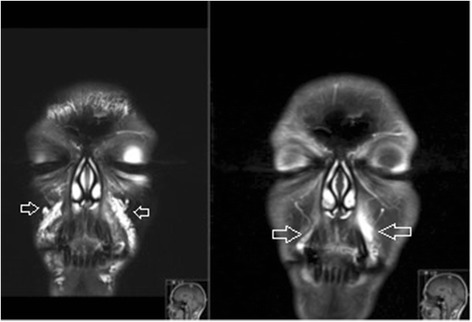
Hyaluronic acid dermal filler in the lower infraorbital triangles (*open arrows*) on coronal MRI (T2FS)

**Fig. 5:**
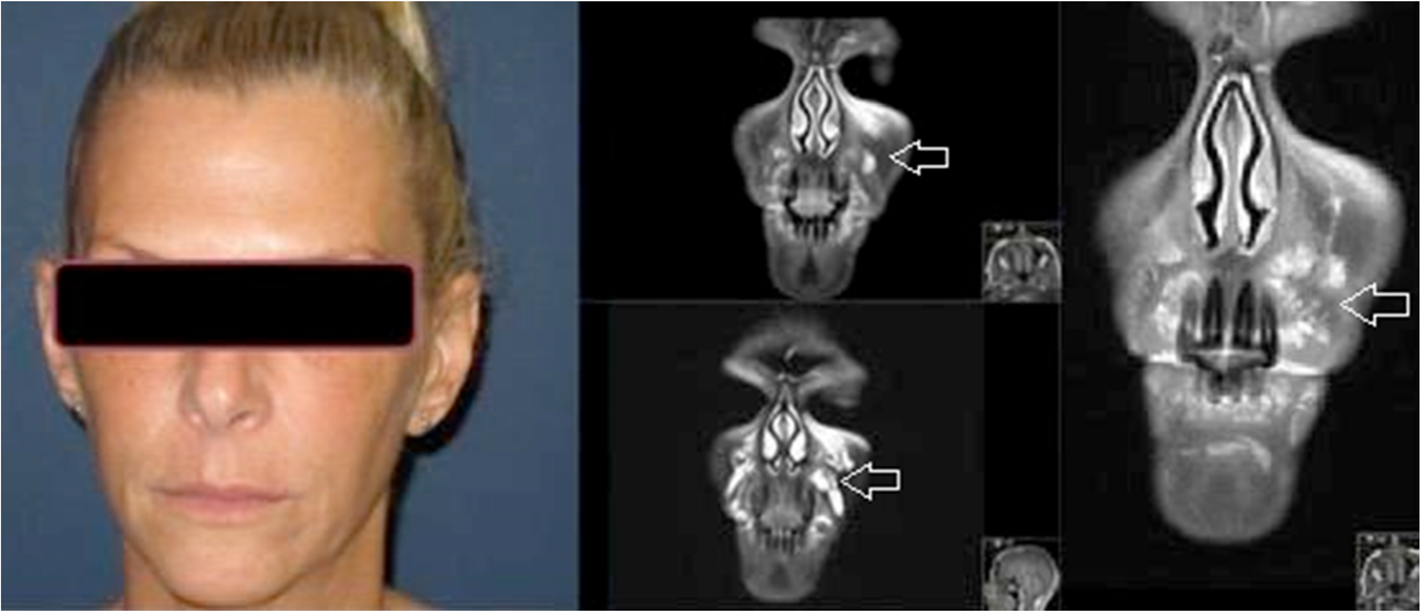
Delayed reaction to calcium hydroxyapetite, coronal MRI (T1FS, T2FS, T1FS with gadolinium). Note the filler in the left infraorbital triangle (*open arrows*), with the delayed reaction extending to the nasolabial sulcus, causing the gross asymetry

For example, when a patient is unsatisfied with the result of a cosmetic injectable filling procedure, MRI could be used to provide an objective evaluation of fullness and symmetry. While scarring, which demonstrates a low signal on T1 weighted images and directly impacts the symmetry of dermal fillers, it does not hamper the correct identification of substance or the radiologist’s ability to assess the distribution of substance. If overfilling is confirmed (Fig. 4), and hyaluronic acid is determined to be the culprit, a simple injection of hyaluronidase can reverse the unwanted outcome [14]. In cases of complications involving an unknown product, imaging can help to establish the identity and guide the approach to treatment. When a granuloma arises from permanent filler (Fig. 5), treatment may require administration of 5-fluoruracil or allopurinol; otherwise, steroids and imiquimod may be used [15, 16]. This further underscores the importance of identification of the precise substance prior to initiating therapy as regards patients’ treatment outcomes.

**Fig. 6:**
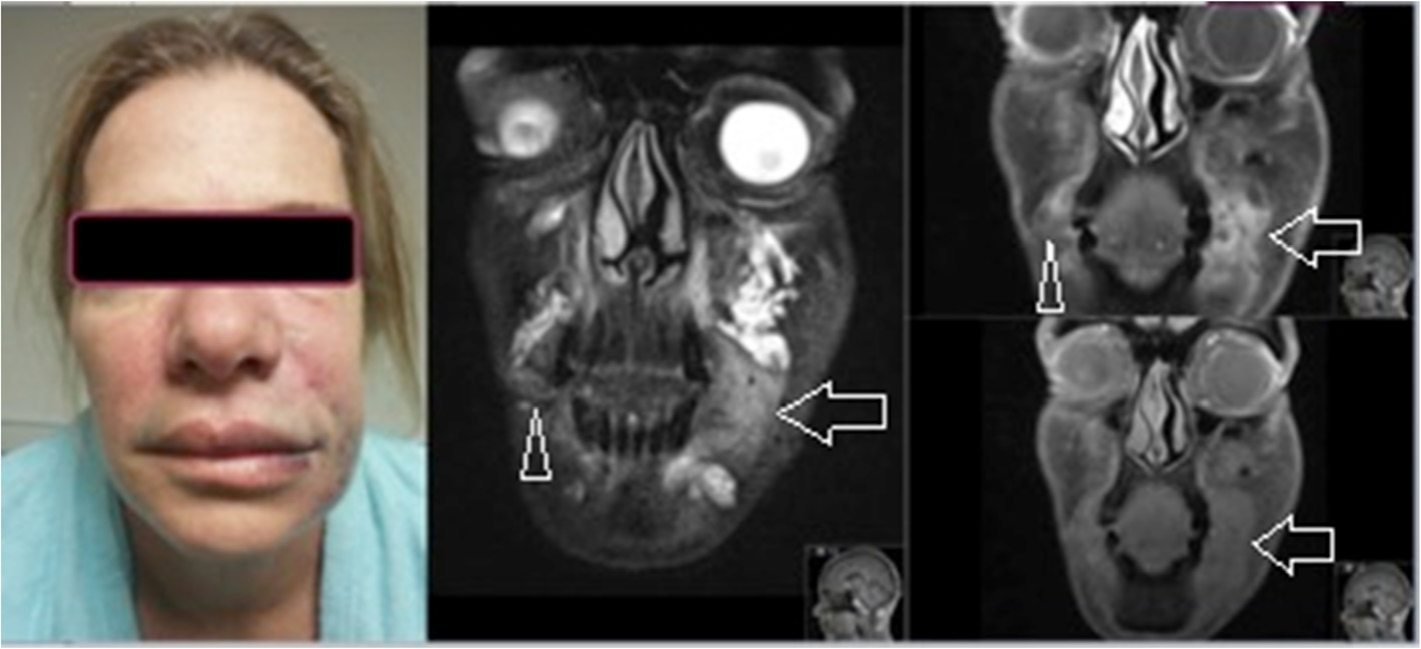
Acute allergic reaction to hyaluronic acid in the left nasolabial sulcus and mouth angle sulcus region (*open arrows*) on coronal MRI (T2FS, FS T1FS with gadolinium). This soft tissue reaction is often reffered to “cellulitis-like.” Note the normal filler in the right lower infraorbital triangle and nasolabial sulcus (*arrowhead*)

In the United States, “any physician who has participated in the care of a patient can be named in a lawsuit if a case has been brought to court” [17]. Unlike in Europe, the prevailing party in frivolous cases cannot recover any legal costs from the losing party [17]. In order to minimize the risk of incurring significant losses of time and money, physicians who administer facial fillers and treat related complications should consider using imaging to validate procedural outcomes. It is recommended that before providing cosmetic care to a patient, all physical findings be photographically documented [18]. MRI could be implemented as an adjunct technique to evaluate asymmetry, overfilling, and true complications, and determine whether the use of dermal fillers has a causal relationship to any pertinent physical findings (Fig. 6). The utility of dedicated facial MRI should be stressed in cases of medico-legal concerns, especially in light of the absence of ionizing radiation and negligible gadolinium risk in healthy patients [19].

The main limitation of our study is sample size. There are other types of facial filler substances on the market than are represented in the sample, and we have not yet had the opportunity to characterize them using our novel MRI technique. Several non-face dedicated techniques have been published in the literature [5, 7], and we did not perform a randomized trial to compare our technique to others; however, the robustness of the Dixon sequences used, which include fat suppression and high resolution field of view, alongside the added value of gadolinium injection, serves as a precise and accurate all-encompassing facial imaging technique. The imaging and clinical utility of this technique was highlighted by our high inter-rater statistics and exquisite images.

Our reference table will be updated each time we encounter cases of complications involving substances not yet seen. Given the growing awareness among referring physicians of the value of dedicated facial MRI with gadolinium injection, we expect to see rising numbers of MRIs performed on cases with and without complications after cosmetic intradermal injectable filler procedures, to evaluate for symmetry and help referring clinicians to provide their post-filler patients with the best possible health and esthetic outcomes.

## Conclusion

**Fig. 7:**
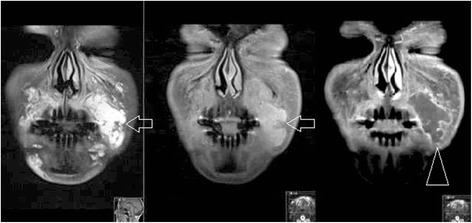
Abscess formation in the left nasolabial sulcus region (*open arrows*) following injection of polyacrylamide, coronal MRI (T2FS, T1FS and T1FS after injection of gadolinium). Note the peripheral “ring-like” enhancement after administration of gadolinium, typical of an abscess (*arrowhead*)

Our research has shown that MRI can be used to determine the identity of unknown facial fillers, and to detect complications including overfilling, asymmetry, abscesses (Fig. 7), and granulomata. MRI imaging can help to guide corrective treatment, to evaluate procedural outcomes, and to provide objective evidence when legal disputes arise. Our study size is small and non-exhaustive; we will continue to add substances to our reference guide as we encounter them.

## Abbreviations

MRI, magnetic resonance imaging; IRB, institutional review board; US, United States; UK, United Kingdom
